# Comprehension of syntax and grammar in dementia

**DOI:** 10.1002/alz70857_106883

**Published:** 2025-12-26

**Authors:** Anusha Ayyar, Shivashankar N, Mathew Varghese, Mariamma Philip

**Affiliations:** ^1^ Octave Speech Centre, Bengaluru, Karnataka, India; ^2^ National Institute of Mental Health And Neurosciences, Bengaluru, Karnataka, India; ^3^ St John's Medical College, Bengaluru, Karnataka, India; ^4^ National Institute of Mental Health and Neurosciences, Bengaluru, India

## Abstract

**Background:**

Sentence comprehension involves the interaction of various complex linguistic (syntactic and grammatical structures) as well as non‐linguistic processes (working memory). There is a lack of consensus among research studies regarding the presence of sentence comprehension deficits in dementia as well as the underlying causes. The present study was conducted to investigate the specific nature and causes of breakdown in sentence comprehension abilities in Kannada (Dravidian Indian language) speaking persons with dementia.

**Method:**

103 persons with mild and moderate dementia [Alzheimer's Disease (*n* = 55); Vascular dementia (*n* = 31); and Frontotemporal dementia (*n* = 17)] as well as 64 normal age matched subjects participated in the study. The effect of sentence length and complexity was assessed using the ‘Revised Token Test ‘(RTT‐Kannada). Grammaticality judgement was evaluated using the ‘Linguistic Profile Test’ (LPT‐Kannada). Baseline scores of cognitive abilities were ascertained using the `Hindi Mental Scale Examination’ (HMSE).

**Result:**

ANOVA and post‐hoc tests revealed a statistically significant difference (*p* <0.001) between the control group and dementia group (mild and moderate) on the total score of RTT and LPT as well as on the subsections. In the RTT, a declining trend in performance from R1 to R10 (increasing sentence length and complexity) was observed in all the 3 groups, with a steeper decline in the moderate dementia group. In the LPT, the most significant deficits were observed for conditional clauses and participle constructions. A qualitative response analysis was also carried out.

Pearson's correlation coefficient showed a strong positive correlation (*p* <0.001) between the syntactic comprehension tests (LPT and RTT) as well as between the HMSE scores and syntactic processing skills.

**Conclusion:**

The results of the study demonstrated the presence of syntactic and grammatical processing deficits in dementia. The study highlights that grammatical incompetence could suggest a genuine linguistic deficit whereas sentence structure analysis through post interpretation, could place higher demands on working memory. Hence cognitive resource deficits could also be a possible underlying cause for the syntactic comprehension deficits. Further studies are required to explore the interaction of cognitive processes and syntactic comprehension deficits.

